# Changing characteristics of hospital admissions but not the children admitted—a whole population study between 2000 and 2013

**DOI:** 10.1007/s00431-017-3064-z

**Published:** 2017-12-19

**Authors:** Maryam Al-Mahtot, Rebecca Barwise-Munro, Philip Wilson, Steve Turner

**Affiliations:** 1 Child Health, Royal Aberdeen Children’s Hospital, Aberdeen, AB25 2ZG UK; 2 Centre for Rural Health, University of Aberdeen, Inverness, UK

**Keywords:** Child, Epidemiology, Hospitalisation

## Abstract

There are increasing numbers of emergency medical paediatric admissions. Our hypothesis was that characteristics of children and details of their emergency admissions are also changing over time. Details of emergency admissions in Scotland 2000–2013 were analysed. There were 574,403 emergency admissions, median age 2.3 years. The age distribution, proportion of boys and socioeconomic status of children admitted were essentially unchanged. Emergency admissions rose by 49% from 36/1000 children per annum to 54/1000 between 2000 and 2013. Emergency admissions that were discharged on the same day rose by 186% from 8.6/1000 to 24.6/1000. The mean duration of emergency admission fell from 1.7 to 1.0 days. The odds for an emergency admission with upper respiratory infection, “viral infection”, tonsillitis, bronchiolitis and lower respiratory tract infection all rose. In contrast the odds for an emergency admission with asthma and gastroenteritis fell.

*Conclusions*: The demographics of children with emergency admissions have not changed substantially but characteristics of admissions have changed considerably, in particular admissions which are short stay and due to respiratory infection are much more common. The fall in the absolute number of children with some acute medical diagnoses suggests that the rise in admissions is not necessarily inexorable.

**Table:** 

**What is Known:** • *Emergency admission prevalence is rising in many countries across Europe.*
**What is New:** *• Our paper is the first to comprehensively analyse emergency medical paediatric admissions by exploring how characteristics of admissions and the children admitted have changed over time for a whole population.* *• The “take home message” is that whilst characteristics of emergency admissions have changed (*e.g. *number, duration of stay, readmissions, diagnoses), the characteristics of the children have not changed.*

**Electronic supplementary material:**

The online version of this article (10.1007/s00431-017-3064-z) contains supplementary material, which is available to authorized users.

## Introduction

The rate of childhood emergency admissions to hospital with acute medical diagnoses in England rose by 28% between 1999 and 2010 [9]. Emergency admissions include those where a child has an unscheduled admission to a hospital ward, and does not include cases seen and discharged home after an emergency department attendance. In the UK, emergency admissions occur when a child is admitted to a hospital inpatient ward after having been referred to medical paediatric services by a doctor or specialist nurse working in either the community (general practice or the primary care out of hours service) or in the hospital emergency department.

The factors driving the rise in paediatric emergency admissions are multifactorial [9], and include patient (or parent) health seeking behaviour, the capacity and expertise for child health care delivery in the community and management in hospital. The rise in paediatric emergency admissions before 2006, which was seen in England [9] and also in Scotland [19], seems likely to be at least in part explained by the 2004 change to the general practice contract leading to near-universal loss of GPs’ responsibility for out-of-hours services for their patients [6] and a 4-hour cap on waiting time in accident and emergency units. The emergence of short stay paediatric assessment units in the late 1990s may have reduced costs but may also have led to increasing emergency admissions [4, 14].

What is not clear is whether the rise in emergency admissions is due to a uniform increase across all children and all diagnoses or whether there are some groups of children and certain diagnoses where admission is becoming more prevalent. Understanding this issue is a prerequisite for the design of any intervention to halt or even reverse the rising number of emergency admissions. In Scotland, the proportion of emergency admissions accounted for by ten “primary care sensitive [16]” diagnoses has remained constant [19] whilst the absolute number of children with these presentations rose between 1999 and 2011 [19]. In England, on the other hand, there was an absolute rise of 20–40% between 1999 and 2010 in the number of emergency admissions with infections, including upper respiratory tract infection [9], lower respiratory tract infection [9], urinary tract infection [9], gastroenteritis [9] and bronchiolitis [10]. A second study which was published by the Nuffield Trust in 2017 of emergency admissions in England and Wales between 2005/6 and 2015/16 reported that overall emergency admissions for those aged up to 24 years rose by 14%, and emergency admissions with viral infection, bronchiolitis and intestinal infection doubled with asthma admissions falling by 20%. Similar trends are seen outside the UK, for example in Sweden there was a doubling in the number of infants admitted with lower respiratory tract infection between 1987 and 2000 [3] and in Denmark there was more than a 50% increase in under 5-year olds admitted with infections between 1980 and 2001 [12]. Previous studies [9, 19] have analysed publicly available data which are limited due to data protection reasons and this limits analysis of admission data to descriptive trends where, for example, covariates cannot be considered.

We have obtained very detailed administrative data for all paediatric emergency admissions in Scotland. Our individual patient analysis allowed us to carry out a comprehensive analysis of emergency admission data whilst adjusting for covariates. Here we extend the previous work in this area [9, 19] by describing trends in emergency admissions and testing the hypothesis that characteristics of patients admitted and details of emergency admissions have changed over time.

## Materials and methods

### Study design

Anonymised details of all admissions to Scottish hospitals for individuals aged ≤ 16 years between January 1, 2000 and December 31, 2013 were provided by the Information Services Division (ISD) of the Scottish Government. In this study, an emergency admission was defined as an episode where a child has an unscheduled admission to a hospital ward. Non-emergency admissions to all medical specialties (e.g. planned admissions for endoscopy or for chemotherapy) and emergency and non-emergency admissions to non-medical specialties (e.g. general surgery, orthopaedic surgery, ear nose and throat surgery) were identified from codes and removed. Children seen and discharged from accident and emergency departments were not included. Children seen by a paediatrician in a scheduled clinic and found to be acutely unwell and admitted for ongoing care were included. In Scotland there are no hospital “walk in” clinics where acutely unwell children are assessed by paediatricians. Each region in Scotland has an emergency department staffed by emergency department staff and which see all patients of ages, there are also three specialist paediatric emergency departments in Scotland which are staffed by emergency department staff but occasionally a member of paediatric staff may be present. Details provided by ISD included the primary diagnosis, age (in years and months), sex, diagnosis, socioeconomic status (Scottish Index of Multiple Deprivation, SIMD, where the population is evenly distributed across quintiles and where 1 is the least affluent quintile), the duration of admission (in whole days) and the health board where the admission took place (Scotland has 14 health boards each covering a distinct geographical area). A unique personal identifier allowed linkage of multiple admissions for an individual. Details of the month and year of each emergency admission were provided allowing cases readmitted within the same calendar month but not within one month after discharge. The following variables were derived: zero day emergency admission (i.e. emergency admission discharged on the same day as admitted and no readmission in the same calendar month); readmission with any diagnosis (i.e. > one emergency admission in the same calendar month regardless of diagnosis); and readmission with the same diagnosis (i.e. > one emergency admission in the same calendar month with the same diagnosis). A complete list of the data available is presented in the on line supplement. The study was approved by the North of Scotland Research Ethics Committee and the ISD Caldicott guardian. Data were stored and analysed in the Grampian data safe haven; the governance for this facility ensures that data cannot be disseminated and any output is carefully vetted to ensure confidentiality prior to release.

### Study outcomes

The International Classification of Diseases-10 codes used for each diagnosis are described in the on line supplement (Table 1). The ten diagnoses (or composite diagnoses) were identified after initially identifying all ICD-10 codes where there were more than 1000 admissions and then, where appropriate creating composite diagnoses where there multiple codes for a similar common diagnosis, e.g. “lower respiratory tract infection” consisted of 17 codes including  J13X pneumonia due to *Streptococcal pneumoniae*, J14X pneumonia due to *Haemophilus pneumoniae*, and also to accommodate shifts in coding, e.g. from K52.9 non-infectious gastroenteritis and colitis to A09.9 Infectious gastroenteritis and colitis unspecified [18]. To establish whether some conditions were becoming more prevalent among zero day admissions and cases readmitted, the ten most prevalent diagnoses (or composite diagnoses) were also identified. The duration of stay was defined as the number of days between the date of admission and date of discharge. In addition to details of diagnoses, the following outcomes were obtained for each calendar year: mean and median age on admission; the total number of admissions; the mean duration of stay (admissions > 14 days long were recoded as 14 days to prevent a small number of very long admission skewing the data); the number of zero day admissions; the number of readmissions with any diagnosis; the number of readmissions with the same diagnosis.

### Hospital episode statistics

To allow comparison with England, the number of emergency admissions and the duration of stay were obtained from Hospital Episode Statistics (HES). The number of emergency admissions to Paediatrics (under Admitted Patient Care: Main Specialties, coded 420) was recorded. The number of emergency admissions in England and Scotland was standardised for the population size for mid-2000 to mid-2013 using data from the Office of National Statistics (ONS). Emergency admission numbers in HES and population size from ONS are presented for the 12 months ending 31 March whereas the number of emergency admissions in Scotland was presented per calendar year and so, for example, in this study HES data from 2000/1 were compared to 2000 data from Scotland.

### Analysis

Logistic regression models were created to describe the change in the proportion of emergency admissions, relative to all admissions, with any of the top ten diagnoses admitted in 2000–2013. The logistic models compared the periods 2000–2003 and 2010–2013 in recognition of the potential for the relationship per annum to be non-linear and that the policy changes in 2003 and 2004 might have led to changes in emergency admission and that 2010–2013 would allow direct comparison one decade later. Year-on-year trends for changes in the proportion of emergency admissions by condition were also derived. The models included sex, deprivation (SIMD), age and month of emergency admission. Additionally, the patient identifier and the hospital region were included to consider clustering of emergency admissions in an individual child and/or an individual hospital. Finally, in order to determine whether there was a shift over time for there to be a change in individual ICD-10 codes within a diagnostic cluster, logistic regression models were created to describe the change in odds for a given ICD-10 code per annum for conditions with more than 1000 admissions. Standard statistical software was used (IBM SPSS version 23.0.0.0). *P* values were not presented due to the very large sample size.

## Results

### Study subjects

**Fig. 1:**
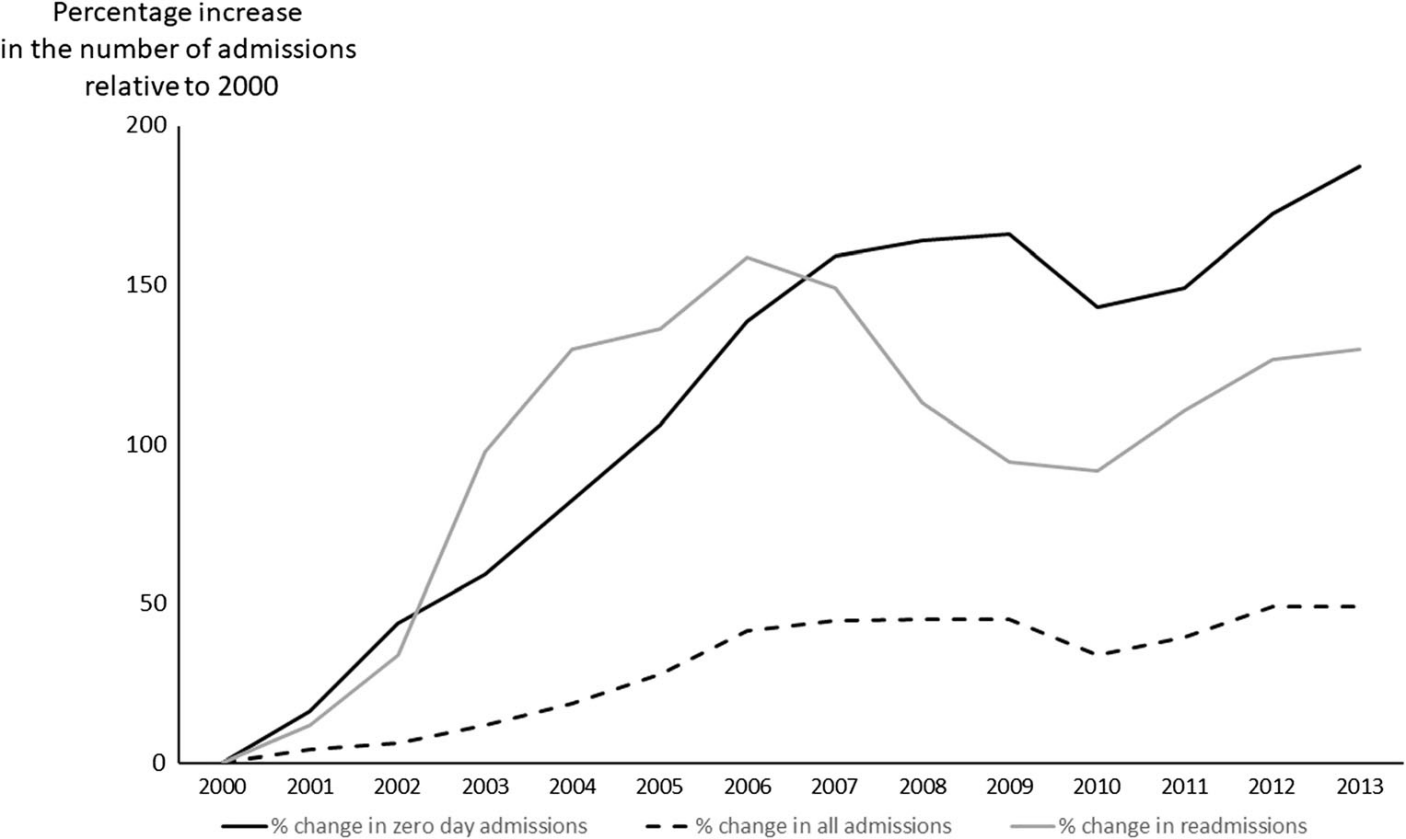
Percentage increase relative to 2000 in the total number of children with an emergency admission, the number with an emergency admission which were discharged on the same day as admitted (i.e. zero day admissions) and the number with more than one admission in a calendar month (readmissions)

**Fig. 2:**
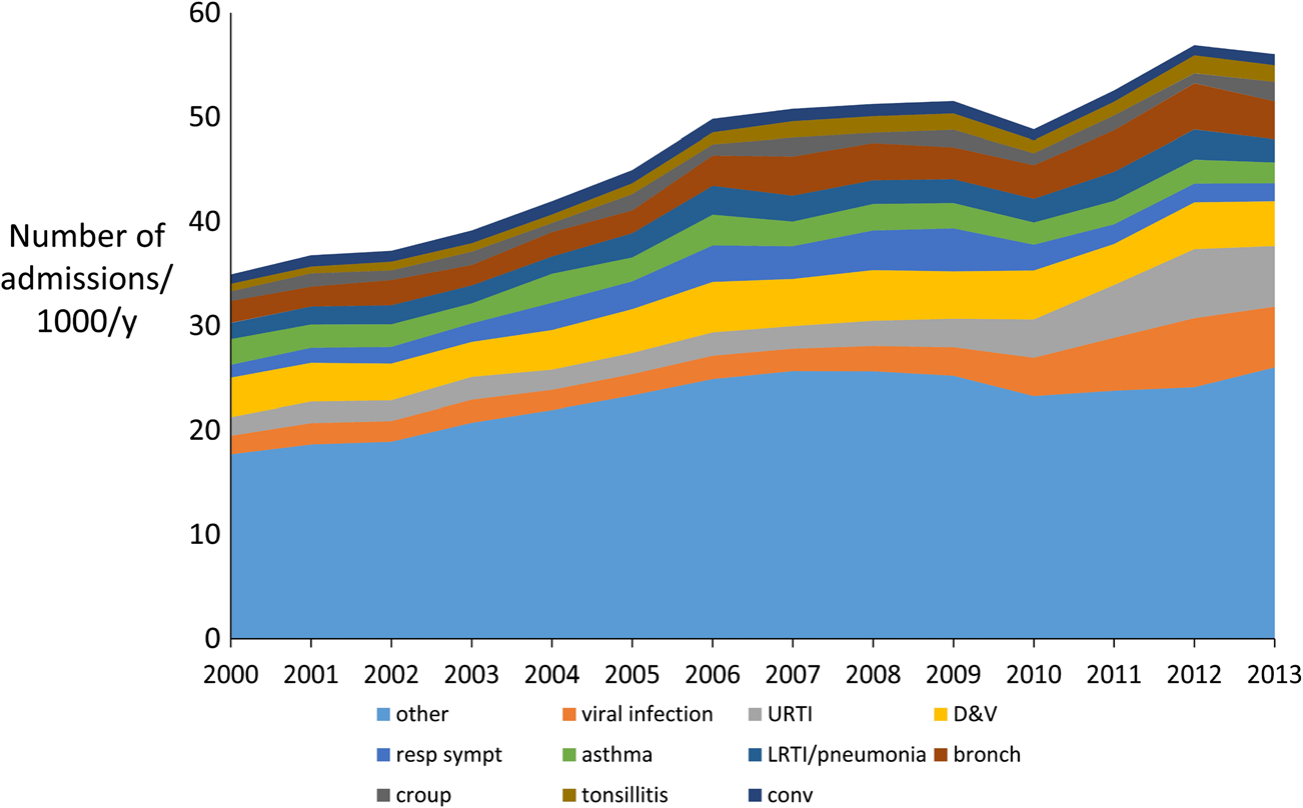
Diagram showing the total number of children with emergency admissions each year, stratified by the ten most common conditions. Other = conditions not in the ten most common. URTI = upper respiratory tract infection, D&V = gastroenteritis, “resp symptoms” = isolated cough, wheeze or shortness of breath, LTRI/pneumonia = lower respiratory tract infection, bronch = bronchiolitis, “conv” = afebrile convulsion

There were 830,705 admissions including 201,457 planned and non-emergency medical paediatric admissions (e.g. endoscopy, chemotherapy) and 58,845 admissions to non-paediatric specialties (e.g. general surgery) leaving 570,403 emergency medical paediatric admissions between 2000 and 2013 for analysis. The median age on emergency admission was 2.3 years [interquartile range 0.8, 6.3]. The rate of emergency admissions rose from 36/1000 in 2000 to 54/1000 in 2013 (Table 1), Fig. 1; corresponding figures for England were 43/1000 and 58/1000. For infants, the prevalence of emergency admissions rose by 37% from 180/1000 in 2000 to 247/1000 in 2013 and similar magnitude % rises were seen in all age groups (Table 2 in supplement). The proportion of boys was 56% and this remained constant throughout. There was a socioeconomic gradient, which was also unchanged throughout, where children from the least affluent quintile (SIMD 1) were over represented (27% of all emergency admissions) whilst the proportions from SIMD 2 to 5 were 22, 19, 17 and 15%, respectively. The ten most common diagnoses accounted for 51.3% of all emergency admissions and were gastroenteritis, upper respiratory tract infection (URTI), viral infection, cough, wheeze or shortness of breath, asthma, lower respiratory tract infection, bronchiolitis, croup, tonsillitis and afebrile convulsion, Fig. 2. Table 1 in the supplement displays the number of cases for each individual ICD-10 code.

**Table 1:** The number of children with an emergency admission each year stratified by zero day and non-zero day admission. Data are presented as absolute numbers, number per 1000 population and percentage change in number per 1000 population with reference to 2000 (ref = reference)

	Total number admitted	Zero day admissions	Non-zero day admissions	Scottish paediatric population	Total number admitted/1000	Zero day admissions/1000	Non-zero day admissions /1000	%change all admissions	%change zero day admissions	%change non-zero admissions
2000	33,305	7862	25,443	919,439	36.22	8.55	27.67	ref	ref	ref
2001	34,097	8995	25,102	904,997	37.68	9.94	27.74	4.01	16.24	0.23
2002	34,308	10,937	23,371	890,242	38.54	12.29	26.25	6.39	43.67	− 5.13
2003	35,504	11,957	23,547	877,685	40.45	13.62	26.83	11.67	59.32	− 3.05
2004	37,467	13,599	23,868	871,907	42.97	15.60	27.37	18.63	82.40	− 1.08
2005	40,042	15,252	24,790	865,091	46.29	17.63	28.66	27.78	106.18	3.55
2006	43,927	17,458	26,469	856,083	51.31	20.39	30.92	41.65	138.49	11.73
2007	44,635	18,851	25,784	851,334	52.43	22.14	30.29	44.74	158.96	9.45
2008	44,744	19,198	25,546	850,206	52.63	22.58	30.05	45.29	164.07	8.58
2009	44,720	19,340	25,380	850,477	52.58	22.74	29.84	45.16	165.94	7.84
2010	41,325	17,699	23,626	851,621	48.53	20.78	27.74	33.96	143.05	0.25
2011	43,204	18,188	25,016	853,891	50.60	21.30	29.30	39.68	149.10	5.87
2012	46,139	19,849	26,290	853,009	54.09	23.27	30.82	49.32	172.13	11.38
2013	46,986	21,332	25,654	868,921	54.07	24.55	29.52	49.28	187.11	6.69

### Changes in relative prevalence of individual diagnoses

In 2013, there were 13,681 more emergency admissions compared to 2000. Between 2000 and 2013, the odds for emergency admissions with URTI fell year-on-year and the odds for an emergency admission diagnosed as viral infection rose. When these two diagnoses were combined (see supplementary Table 3), the proportion of all emergency admissions for URTI or viral infection rose from 13.6% for 2000–2003 to 17.0% for 2010–2013, equivalent to an increase of 29% [26, 31]. There were 3956 more emergency admissions with URTI and viral infection in 2014 compared to 2000, Table 2. There were reductions in the relative proportion of emergency admissions with diarrhoea and vomiting, asthma, croup, afebrile convulsion, constipation and isolated cough, wheeze or shortness of breath, Table 1 and supplemental Table 4. When individual ICD-10 coded conditions within composite diagnoses were analysed separately, trends were unsurprisingly similar to the trend for the composite diagnosis (supplemental Table 1).

**Table 2:** Odds ratio for having an emergency admission with one of the ten most common diagnoses (2000–2013) for the period 2010–2013 relative to 2000–2013. Odds ratios were adjusted for age, sex, deprivation, month of admission and clustering of admissions for an individual child and individual health board

Diagnoses	Total number of emergency admissions with the condition	Emergency admissions per 1000 children	Proportion of all emergency admissions	Odds ratio for emergency admission comparing 2010–2013 with 2000–2003
Gastroenteritis	50,866	4.18	8.8%	0.863 [0.842, 0.885]
Upper respiratory tract infection	41,921	3.45	7.3%	0.777 [0.756, 0.798]
Viral infection	36,910	3.03	6.5%	2.060 [2.002, 2.120]
Bronchiolitis	35,769	2.94	6.3%	1.240 [1.102, 1.279]
Cough, wheeze or shortness of breath	29,156	2.40	5.1%	0.932 [0.898, 0.967]
Asthma (not including wheeze)	28,521	2.34	5.0%	0.732 [0.708, 0.757]
Lower respiratory tract infection (including pneumonia)	26,626	2.19	4.7%	1.097 [1.061, 1.135]
Croup (not including stridor)	15,381	1.19	2.7%	0.876 [0.838, 0.915]
Tonsillitis	14,427	1.13	2.5%	1.460 [1.392, 1.531]
Convulsion	13,715	4.18	2.4%	0.734 [0.697, 0.765]

### Change in relative prevalence of diagnoses among zero day admissions and readmissions

There were 220,517 zero day admissions and the ten most common diagnoses were as follows (beginning with the most common): URTI/viral infection, gastroenteritis, croup, bronchiolitis, tonsillitis, asthma, non-specific abdominal pain, constipation, febrile convulsion and rash (supplemental Table 5). These ten conditions contributed approximately 50% of all zero day admissions between 2000 and 2013. There was evidence of a modest rise in the proportion of zero day admissions due to URTI/viral infection, bronchiolitis and tonsillitis and a most fall in diarrhoea and vomiting and asthma (supplemental Table 5). Among the 43,557 cases readmitted with the same diagnosis within the same month, the ten most common diagnoses were as follows (starting with the most common): bronchiolitis, URTI/febrile illness, gastroenteritis, non-specific respiratory symptoms, croup, asthma, gastro-oesophageal reflux, constipation, fever and febrile convulsion (supplemental Table 6). These conditions explained approximately 30% of readmissions. There was a rise in the proportion of readmissions with bronchiolitis and croup and falls in the proportion with gastroenteritis and asthma (supplemental Table 6).

### Changes in characteristics of admission

The mean duration of stay was 1.7 days in 2000, 1.1 days in 2007 and 1.0 day in 2013, Fig. 1, supplemental Table 7. The rate of zero day emergency admissions rose from 9/1000 to 25/1000 between 2000 and 2013 (or by 189%) between 2000 and 2013, Fig. 1; there were 13,478 more zero day emergency admissions in 2013 compared to 2000. The proportion of all emergency admissions that were zero day admissions was 25% in 2000 and 50% in 2013 (Table 8 online supplement). The proportion of all children readmitted with any diagnosis rose from 5.4% in 2000 to 8.5% in 2005 and remained roughly stable thereafter and was 8.2% in 2013; a similar pattern was seen for readmissions with the same diagnosis with corresponding figures of 2.6, 4.8 and 4.0%, Fig. 1, supplemental Table 8. The median age of children changed marginally with an apparent step-wise change between 2006 and 2007; the median age of children was 2.4 years between 2000 and 2006 and 2.1 between 2007 and 2013. The proportion of infants admitted was highest (33.2%) in 2008 and lowest in 2003 (28%), Table 2 online supplement.

## Discussion

To our knowledge this is the most comprehensive assessment of paediatric emergency admissions to hospital for a whole nation. Our study has described trends in all emergency admissions, zero day emergency admissions, readmissions and also the diagnoses and characteristics of the children admitted. Our work is the first consider covariates in the analyses, meaning that our results are independent of the child’s socioeconomic status, sex and age and also any difference in practice between hospitals. The main finding was that over time, the characteristics of the children with an emergency admission were constant but there were obvious differences in the details of the admissions. There has been considerable change in the number and profile of emergency admissions over a short period of time, and these changes are highly likely to continue and place further burden on the healthcare system in Scotland, across the whole of the UK and other European countries, despite differences in health care infrastructures. For example, there are rising admissions of young children with acute infections in Scandinavia [3, 12] and in Italy there is concern that a considerable minority of admissions may be avoidable [2].

Rising emergency admissions in the UK have been attributed to a “systematic failure, both in primary care….and in hospital…..in the assessment of children with acute illness [9]” however reductions in the relative proportion and absolute number of children admitted with some chronic conditions, e.g. asthma and afebrile convulsions, argues that there is capacity to manage children in the community without the need for emergency admissions. The introduction in Scotland of managed clinical networks for paediatric medical specialities in the mid-2000s may be relevant to the fall in emergency admissions with chronic conditions What is not clear is why the number of emergency admissions with some acute conditions has fallen, e.g. croup and gastroenteritis, whereas those for other conditions have risen, e.g. bronchiolitis, lower respiratory tract infection and tonsillitis.

Our results are consistent with results from England described in one published study [9] and the 2017 report from the Nuffield Trust which also finds increasing absolute numbers of children with emergency admissions due to lower respiratory tract infections and falling numbers admitted with asthma. A study from Spain also reports falling asthma admissions in children and young adults [8]. Our study findings are also consistent with a year-on-year rise in bronchiolitis admissions in England [10]. Rises in the number and proportion of emergency admissions lasting less than one [9] or two days [17] have been reported in England up to 2010 and our study demonstrates that this trend continues. Previous work has described a greater increase in emergency admissions among younger relative to older children [9, 15] and whilst we see that the greatest absolute increase in the number of emergency admissions of infants compared to other age groups, the relative increase in emergency admission prevalence was mostly constant across all ages. One result which is not consistent with previous reports is the fall in gastroenteritis emergency admissions in Scotland which is in contrast with a rise England [9]. The mostly comparable trends in the number of acute admissions in England and Scotland plus similar trends in diagnoses made indicate that our results are generalisable outside Scotland.

This study was not designed to explain why the number of emergency admission is rising, but some of the results might give a steer as to factors which do not contribute large numbers of emergency admissions. For example there was divergence in the trends in duration of stay (falling) and emergency admissions (rising) and these trends might be linked if children were being discharged home “too early” only to be readmitted, and whilst there was a rise in readmissions before 2006, this proportion remained stable from that point onwards and the numbers involved were too small to explain a substantial proportion of the rising emergency admissions seen. A second insight is that the change in readmissions was parallel to that of zero day emergency admissions but the former was less than 10% of the latter, and therefore readmissions are not a substantial reason for rising zero day emergency admissions. The rise in emergency admissions was entirely due to zero day admissions and this might reflect a combination of factors such as (i) more rapid assessment and effective treatment of acute presentations, (ii) changes in health seeking behaviour of parents, (iii) declining expertise and resources in the community to “watch and wait” and (iv) risk aversion among admitting clinicians in the context of availability of acute assessment wards.

Overall, the diagnoses for zero day admissions were similar to those for all admissions although non-specific abdominal pain and rash were only seen among the ten most common diagnoses for zero day admissions. The trends for rising numbers and proportion of all admissions with acute respiratory infections and falls in asthma and gastroenteritis were also seen within zero day admissions (supplemental Table 5). The characteristics of diagnoses for readmissions was different to zero day and all admissions in at least two aspects: first the ten most common diagnoses for readmission explained a smaller proportion (30%) of all readmissions compared to the corresponding proportion for zero day admissions (50%); second the proportion of readmissions with different diagnoses remained roughly unchanged with the exception of bronchiolitis (which rose). These observations suggest that there are different drivers for zero day admission and readmission.

A 2005 review concluded that short stay assessment units might be effective in reducing paediatric emergency admissions [15] but a 2012 systematic review did not confirm this finding and concluded that there was no clear solution to the question “how do we reduce emergency admission?” [7]. An observational study published in 2003 which reported activity in one assessment unit suggested that one third of emergency admissions can be immediately sent home [13] and only 2% were readmitted [14]. Many UK centres now have paediatric assessment wards or short stay units yet the number of children being admitted continues to rise and this suggests that short stay units are not effective in reducing emergency admissions (and might be part of the reason for rising admission). Interventions “outside” the hospital should now be explored. There is little understood about what factors in primary care affect emergency admissions, although one systematic review suggested that continuity of care with a single primary care clinician was important [11]. Other potential interventions could include assessment of the child at the point of referral by specialist via videolink and brief educational interventions delivered to clinical decision-makers focussed on specific conditions.

This study has a number of strengths and limitations. We considered the potential for change in trends of coding to cause an apparent fall or rise in a diagnosis presenting to hospital, and we detected a fall in URTI at the same time as admissions with viral infection rose, and also we used clusters of codes to ensure that, for example, we captured any drift in gastroenteritis coding from R52.9 to A09.9. A limitation is that diagnostic coding is known to be imperfect but is sufficiently robust for research and managerial decision-making [5], and moreover these errors are likely to reduce and not magnify the trends we have described. A second limitation is that some of the change in diagnoses might be due to changes in the true prevalence and not the threshold for emergency admission and the fall in asthma prevalence since 2004 [1] might at least partly explain the fall in asthma emergency admissions we report here. A third limitation is that we were able to identify emergency admissions within the same calendar month of discharge but not within one month per se, anecdotally most readmissions occur within a few days of discharge so this is unlikely to affect many readmissions but might underestimate the true readmission rates. A final limitation is that although the trends reported here are valid for the whole country and we considered “health board” as a covariate, some aspects of patient management are likely differ to some degree across the regions within Scotland.

In summary, our whole population study has identified that within the overall rise in the number of emergency admissions in children, the patient demographics have not changed substantially but details of the emergency admissions have changed considerably over a 14-year period. Understanding trends in conditions leading to emergency admissions can inform interventions aimed at safely arresting and perhaps reversing these trends.

## Electronic supplementary material

ESM 1 (DOCX 49.2 kb)

## Abbreviations

GP: General practitioner
HES: Hospital Episode Statistics
ICD: International Classification of Diseases
ISD: Information Services Division of the Scottish Government
ONS: Office of National Statistics
SIMD: Scottish Index of Multiple Deprivation
URTI: Upper respiratory tract infection
