# Supplementary material for: Changing characteristics of hospital admissions but not the children admitted—a whole population study between 2000 and 2013
Source: Eur J Pediatr. 2017 Dec 19;177(3):381–8. doi: 10.1007/s00431-017-3064-z (PMC5816774; doi:10.1007/s00431-017-3064-z)
Supplement: Supplementary file 1 — (DOCX 49.2 kb) [file 431_2017_3064_MOESM1_ESM.docx]

Table 1. The International Classification of Diseases-10 codes were used to categorise conditions or condition groups. The table includes coding clusters for the ten most common conditions plus others which were candidates for being in the ten most common. The table also states the number of admissions for each separate ICD-10 code. Changing trends in coding within coding clusters were explored by describing the change in odds ratio for each individual ICD-10 code where there were more than 1000 admissions.

| Condition/condition group | ICD-10 code | Description | Number of admissions | Unadjusted odds ratio for admission with ICD-10 code relative to other diagnoses changing year-on-year with reference to 2000 |
| --- | --- | --- | --- | --- |
| Asthma | J45.0 | Predominantly allergic asthma | 11712 | 0.931 [0.926, 0.935] |
|  | J45.9 | Other and unspecified asthma | 15398 | 1.006 [0.112, 1.010] |
|  | J46X | Status Asthmaticus* | 1411 | 0.778 [0.766, 0.791] |
| Bronchiolitis | J21.0 | Acute bronchiolitis due to respiratory syncitial virus | 17874 | 0.997 [0.993, 1.001] |
|  | J21.8 | Acute bronchiolitis due to other specified organisms | 875 |  |
|  | J21.9 | Acute bronchiolitis unspecified | 16800 | 1.059 [1.054, 1.063] |
|  | J12.1 | Respiratory syncitial virus pneumonia | 220 | 1.032 [0.998, 1.068] |
| Afebrile convulsion | G40.9 | Epilepsy unspecified | 4535 | 0.955 [0.948, 0.962] |
|  | R56.8 | Unspecified convulsions | 9180 | 0.975 [0.970, 0.980] |
| Constipation | K59.0 | Constipation | 9208 |  |
| Croup | J05.0 | Acute obstructive laryngitis [croup] | 15381 |  |
| Gastroenteritis | A08.0 | Rotavirus | 3935 | 0.977 [0.970, 0.985] |
|  | A08.1 | Norwalk virus | 116 |  |
|  | A08.2 | Adenovirus | 323 |  |
|  | A08.3 | Other virus enteritis | 659 |  |
|  | A08.4 | Viral intestinal infection unspecified | 22354 | 0.993 [0.989, 0.996] |
|  | A09.0 | Infectious gastroenteritis and colitis unspecified | 168 |  |
|  | A09.9 | Gastroenteritis and colitis of unspecified origin | 644 |  |
|  | A09X | Infectious gastroenteritis and colitis, unspecified | 2022 | 0.957 [0.947, 0.968] |
|  | K52.9 | Non infectious gastroenteritis and colitis | 20645 | 0.968 [0.965, 0.972] |
| Upper respiratory tract infection | J06.9 | Acute upper respiratory tract infection, unspecified | 40853 | 0.979 [0.977, 0.982] |
|  | J00X | Acute nasopharyngitis | 1068 | 1.113 [1.095, 1.131] |
| Viral infection | B34.9 | Viral infection, unspecified | 36910 | 1.091 [1.088, 1.094] |
| Acute otitis media | H65.0 | Acute serous otitis media | 346 |  |
|  | H66.9 | Otitis media unspecified | 3037 | 0.898 [0.980, 0.997] |
|  | H66.0 | Acute suppurative otitis media | 74 |  |
|  | H66.4 | Suppurative otitis media | 28 |  |
| Tonsillitis | J03.9 | Acute tonsillitis unspecified | 12461 | 1.043[1.038, 1.048] |
|  | J02.9 | Acute pharyngitis, unspecified | 1246 | 1.017 [1.003, 1.031] |
|  | J03.8 | Acute tonsillitis due to other organisms | 219 |  |
|  | J03.0 | Streptococcal tonsillitis | 501 |  |
| Febrile convulsion | R56.0 | Febrile convulsions | 13671 |  |
| Lower respiratory tract infection | J12.0* | Adenoviral pneumonia | 32 |  |
|  | J12.2* | Parainfluenza virus pneumonia | 37 |  |
|  | J12.8* | Other viral pneumonia | 56 |  |
|  | J12.9* | Viral pneumonia unspecified | 207 |  |
|  | J13X* | Pneumonia due to Streptococcus pneumoniae | 155 |  |
|  | J14X* | Pneumonia due to Haemophilus influenzae | 48 |  |
|  | J15.1* | Pneumonia due to Pseudomonas | 18 |  |
|  | J15.2* | Pneumonia due to Staphylococcus | 11 |  |
|  | J15.4* | Pneumonia due to other streptococci | 52 |  |
|  | J15.7* | Pneumonia due to Mycoplasma pneumoniae | 641 |  |
|  | J15.8* | Pneumonia due to other specified bacteria | 20 |  |
|  | J15.9* | Unspecified bacterial pneumonia | 62 |  |
|  | J18.0* | Bronchopneumonia unspecified organism | 350 |  |
|  | J18.1* | Lobar pneumonia, unspecified organism | 5732 | 0.974 [0.968, 0.981] |
|  | J18.1D* | Lobar pneumonia with pleural effusion | 111 |  |
|  | J18.8* | Other pneumonia with effusion | 57 |  |
|  | J18.9* | Pneumonia, unspecified organism | 3543 | 0.966 [0.958, 0.975] |
|  | J22X* | Unspecified acute lower respiratory infection | 15662 | 1.038 [1.034, 1.042] |
| Cough, wheeze or shortness of breath | R05X | Cough | 4819 | 1.049 [1.041, 1.056] |
|  | R06.2 | Wheezing | 17752 | 0.984 [0.981, 0.988] |
|  | R06.0 | Dyspnoea | 1350 | 1.029 [1.015, 1.043] |
|  | R06.8 | Other abnormalities of breathing | 5235 | 0.982 [0.976, 0.989] |

*there were no admissions with more recent codes for status asthmaticus (J45.22, J45.32, J45.52, J45.902)

Table 2. Absolute number (N) of children admitted to hospital and prevalence (P) of admissions/1000 population by year of age per annum.*not calculated due to relatively small numbers admitted in 2000

|  | 2000 | | 2001 | | 2002 | | 2003 | | 2004 | | 2005 | | 2006 | | 2007 | |
| --- | --- | --- | --- | --- | --- | --- | --- | --- | --- | --- | --- | --- | --- | --- | --- | --- |
|  | N | P | N | P | N | P | N | P | N | P | N | P | N | P | N | P |
| Infants | 9599 | 180 | 9256 | 178 | 9726 | 190 | 9370 | 181 | 10584 | 197 | 11130 | 204 | 12406 | 225 | 13109 | 230 |
| 1 -1.9 year olds | 5692 | 102 | 5660 | 107 | 5815 | 111 | 5876 | 115 | 6043 | 116 | 6798 | 126 | 7591 | 139 | 7817 | 141 |
| 2-2.9 year olds | 3534 | 62 | 3747 | 67 | 3685 | 69 | 3930 | 75 | 3679 | 72 | 4044 | 78 | 4419 | 82 | 4614 | 84 |
| 3-3.9 year olds | 2570 | 44 | 2546 | 45 | 2603 | 47 | 2859 | 53 | 2812 | 53 | 2969 | 57 | 3153 | 60 | 3177 | 59 |
| 4-4.9 year olds | 1851 | 32 | 1979 | 34 | 1838 | 32 | 1980 | 35 | 2235 | 41 | 2212 | 42 | 2326 | 45 | 2176 | 41 |
| 5-5.9 year olds | 1416 | 24 | 1566 | 27 | 1564 | 27 | 1550 | 27 | 1607 | 29 | 1744 | 32 | 1835 | 34 | 1835 | 35 |
| 6-6.9 year olds | 1153 | 19 | 1220 | 21 | 1170 | 20 | 1323 | 22 | 1401 | 24 | 1401 | 25 | 1674 | 31 | 1495 | 28 |
| 7-7.9 year olds | 977 | 16 | 1130 | 19 | 1054 | 18 | 1183 | 20 | 1340 | 23 | 1300 | 22 | 1341 | 24 | 1280 | 23 |
| 8-8.9 year olds | 998 | 15 | 1006 | 16 | 972 | 16 | 1007 | 17 | 1202 | 20 | 1312 | 22 | 1312 | 23 | 1204 | 21 |
| 9-9.9 year olds | 1026 | 16 | 1021 | 16 | 970 | 15 | 1036 | 17 | 1064 | 18 | 1259 | 21 | 1318 | 22 | 1224 | 21 |
| 10-10.9 year olds | 926 | 15 | 1063 | 16 | 1022 | 16 | 1039 | 17 | 1098 | 18 | 1086 | 18 | 1326 | 22 | 1297 | 21 |
| 11-11.9 year olds | 935 | 15 | 961 | 15 | 1023 | 16 | 996 | 15 | 1040 | 17 | 1113 | 18 | 1211 | 20 | 1208 | 20 |
| 12-12.9 year olds | 936 | 14 | 1006 | 16 | 977 | 15 | 1123 | 17 | 1152 | 18 | 1171 | 19 | 1323 | 21 | 1217 | 20 |
| 13-13.9 year olds | 891 | 14 | 1037 | 16 | 895 | 14 | 1004 | 16 | 1013 | 15 | 1177 | 18 | 1203 | 19 | 1230 | 20 |
| 14-14.9 year olds | 541 | 8 | 618 | 10 | 667 | 10 | 760 | 12 | 698 | 11 | 798 | 12 | 907 | 14 | 972 | 15 |
| 15-15.9 year olds | 260 | 4 | 281 | 4 | 327 | 5 | 468 | 7 | 499 | 8 | 528 | 8 | 582 | 9 | 780 | 12 |
|  | 2008 | | 2009 | | 2010 | | 2011 | | 2012 | | 2013 | | Absolute change in number admitted 2000-2013 | | Relative change in number admitted 2000-2013 | |
|  | N | P | N | P | N | P | N | P | N | P | N | P |  |  |  |  |
| Infants | 14056 | 236 | 13310 | 223 | 12550 | 212 | 13146 | 218 | 14317 | 245 | 14073 | 247 | 4474 | | 37 | |
| 1 -1.9 year olds | 7861 | 137 | 8176 | 137 | 7411 | 125 | 7472 | 130 | 8152 | 135 | 8381 | 143 | 2689 | | 40 | |
| 2-2.9 year olds | 4546 | 82 | 4564 | 80 | 4378 | 74 | 4526 | 76 | 4749 | 82 | 4794 | 79 | 1260 | | 27 | |
| 3-3.9 year olds | 3102 | 56 | 3326 | 60 | 3070 | 5 | 3270 | 55 | 3768 | 63 | 3597 | 62 | 1027 | | 42 | |
| 4-4.9 year olds | 2218 | 41 | 2440 | 44 | 2061 | 37 | 2305 | 40 | 2610 | 44 | 2564 | 43 | 713 | | 35 | |
| 5-5.9 year olds | 1752 | 33 | 1822 | 33 | 1613 | 29 | 1720 | 31 | 1923 | 34 | 2038 | 34 | 622 | | 43 | |
| 6-6.9 year olds | 1439 | 27 | 1388 | 26 | 1317 | 24 | 1499 | 27 | 1485 | 27 | 1604 | 28 | 451 | | 49 | |
| 7-7.9 year olds | 1142 | 21 | 1307 | 25 | 1158 | 22 | 1201 | 22 | 1241 | 22 | 1282 | 23 | 305 | | 47 | |
| 8-8.9 year olds | 1201 | 22 | 1207 | 22 | 994 | 19 | 1091 | 21 | 1049 | 19 | 1172 | 21 | 174 | | 38 | |
| 9-9.9 year olds | 1123 | 19 | 1149 | 21 | 1083 | 20 | 1089 | 21 | 1014 | 19 | 1139 | 21 | 113 | | 33 | |
| 10-10.9 year olds | 1222 | 21 | 1061 | 18 | 1057 | 19 | 1037 | 19 | 1004 | 19 | 1093 | 21 | 167 | | 42 | |
| 11-11.9 year olds | 1197 | 20 | 1129 | 19 | 979 | 17 | 1057 | 19 | 1011 | 18 | 1076 | 20 | 141 | | 39 | |
| 12-12.9 year olds | 1184 | 20 | 1198 | 20 | 1067 | 18 | 1095 | 19 | 1077 | 19 | 1236 | 23 | 300 | | 58 | |
| 13-13.9 year olds | 1090 | 18 | 1045 | 17 | 1032 | 17 | 1027 | 17 | 998 | 17 | 1042 | 19 | 151 | | 35 | |
| 14-14.9 year olds | 898 | 14 | 877 | 14 | 825 | 14 | 927 | 15 | 905 | 15 | 948 | 16 | 407 | | * | |
| 15-15.9 year olds | 713 | 11 | 721 | 11 | 730 | 12 | 742 | 12 | 836 | 14 | 947 | 16 | 687 | | * | |

Table 3. Proportion of children with diagnosis of upper respiratory tract infection and viral infection per annum. n=number of admissions

|  | Viral infection (n) | Upper respiratory tract infection (n) | Combined % (n) |
| --- | --- | --- | --- |
| 2000 | 4.9% (1632) | 8.5% (2822) | 13.4% (4454) |
| 2001 | 5.4% (1851) | 7.8% (2670) | 13.3% (4521) |
| 2002 | 5.1% (1751) | 8.6% (2944) | 13.7% (4695) |
| 2003 | 5.4% (1914) | 8.6% (3044) | 14.0% (4958) |
| 2004 | 4.5% (1680) | 6.8% (2563) | 11.3% (4243) |
| 2005 | 4.4%(1760) | 7.3% (2920) | 11.7% (4680) |
| 2006 | 4.4% (1917) | 7.2% (3179) | 11.6% (5096) |
| 2007 | 4.1% (1838) | 7.2% (3226) | 11.3% (5064) |
| 2008 | 4.6% (2064) | 7.2% (3224) | 11.8% (5288) |
| 2009 | 5.2% (2327) | 7.2% (3207) | 12.4% (5534) |
| 2010 | 7.6% (3130) | 6.9%(2857) | 14.5% (5987) |
| 2011 | 10.0% (4330) | 6.2% (2662) | 16.2% (6992) |
| 2012 | 12.3% (5654) | 7.1% (3255) | 19.3% (8909) |
| 2013 | 10.8% (5062) | 7.1% (3348) | 17.9% (8410) |

Table 4. Odds ratio for a child being admitted with a given condition year on year between 2000 and 2013. Odds ratios were adjusted for age, sex, deprivation, month of admission. To consider the potential for clustering of admissions for a child or a hospital, the unique identifier for each child and the health board where the admission took place were also included as covariates.

| Diagnoses | Total number of admissions with the condition | Odds ratio for admission relative to other diagnoses changing year-on-year |
| --- | --- | --- |
| Gastroenteritis | 50,866 | 0.984 [0.982, 0.986] |
| Upper respiratory tract infection | 41,921 | 0.979 [0.977, 0.982] |
| Viral infection | 36,910 | 1.090 [1.087, 1.093] |
| Bronchiolitis | 35,769 | 1.022 [1.019, 1.025] |
| Cough, wheeze or shortness of breath | 29,156 | 0.993 [0.990, 0.996] |
| Asthma (not including wheeze) | 28,521 | 0.965 [0.962, 0.968] |
| Lower respiratory tract infection (including pneumonia) | 26,626 | 1.007 [1.004, 1.010] |
| Croup (not including stridor) | 15,381 | 0.993 [0.989, 0.997] |
| Tonsillitis | 14,427 | 1.042 [1.037, 1.046] |
| Convulsion | 13,715 | 0.969 [0.965, 0.973] |

Table 5. The absolute number of zero day admissions stratified by the top ten diagnoses for zero day admissions between 2000 and 2013. The proportion of zero day admissions with each diagnosis are also presented in brackets. NSAP=non-specific abdominal pain (ICD-10 code R14.0). Rash was defined as an ICD-10 code R21X.

|  | total of all zero day  (% with ten most common diagnoses) | URTI/viral infection | Gastroenteritis | Croup | Asthma | Bronchiolitis | Febrile convulsion | Constipation | Tonsillitis | NSAP | Rash |
| --- | --- | --- | --- | --- | --- | --- | --- | --- | --- | --- | --- |
| 2000 | 7862  (50%) | 1286  (16%) | 852  (11%) | 386  (5%) | 319  (4%) | 226  (3%) | 202  (3%) | 173  (2%) | 172  (2%) | 172  (2%) | 110  (1%) |
| 2001 | 8995  (48%) | 1393  (15%) | 881  (10%) | 545  (6%) | 335  (4%) | 238  (3%) | 247  (3%) | 220  (2%) | 169  (2%) | 182  (2%) | 110  (1%) |
| 2002 | 10937  (49%) | 1862  (17%) | 1080  (10%) | 449  (4%) | 418  (4%) | 347  (3%) | 284  (3%) | 273  (2%) | 281  (3%) | 196  (2%) | 159  (1%) |
| 2003 | 11957  (50%) | 2149  (18%) | 1058  (9%) | 560  (5%) | 386  (3%) | 343  (3%) | 372  (3%) | 330  (3%) | 327  (3%) | 270  (2%) | 203  (2%) |
| 2004 | 13599  (48%) | 2036  (15%) | 1278  (9%) | 459  (3%) | 641  (5%) | 512  (4%) | 321  (2%) | 320  (2%) | 339  (2%) | 357  (3%) | 280  (2%) |
| 2005 | 15252  (49%) | 2369  (16%) | 1523  (10%) | 773  (5%) | 532  (3%) | 447  (3%) | 403  (3%) | 387  (3%) | 446  (3%) | 306  (2%) | 252  (2%) |
| 2006 | 17458  (49%) | 2765  (16%) | 1927  (11%) | 559  (3%) | 657  (4%) | 659  (4%) | 482  (3%) | 375  (2%) | 478  (3%) | 352  (2%) | 322  (2%) |
| 2007 | 18851  (51%) | 2843 (15%) | 1931  (10%) | 861  (5%) | 589  (3%) | 971  (5%) | 491  (3%) | 369  (2%) | 731  (4%) | 423  (2%) | 329  (2%) |
| 2008 | 19198  (49%) | 3082 (16%) | 2051 (11%) | 514  (3%) | 633  (3%) | 894  (5%) | 490  (3%) | 303  (2%) | 727  (4%) | 404  (2%) | 343  (2%) |
| 2009 | 19340  (49%) | 3240 (17%) | 1866  (10%) | 817  (4%) | 549  (3%) | 774  (4%) | 393  (2%) | 324  (2%) | 760  (4%) | 376  (2%) | 372  (2%) |
| 2010 | 17699  (50%) | 3161 (18%) | 1989 (11%) | 591  (3%) | 508  (3%) | 776  (4%) | 313  (2%) | 297  (2%) | 648  (4%) | 372  (2%) | 238  (1%) |
| 2011 | 18188  (50%) | 3540 (19%) | 1610 (9%) | 701  (4%) | 541  (3%) | 964  (5%) | 300 (2%) | 304  (2%) | 613  (3%) | 351  (2%) | 237  (1%) |
| 2012 | 19849  (53%) | 4334 (22%) | 1922 (10%) | 475  (2%) | 541  (3%) | 1138  (6%) | 307  (2%) | 358  (2%) | 825  (4%) | 328  (2%) | 258  (1%) |
| 2013 | 21332  (50%) | 4239 (20% | 1834  (9%) | 921  (4%) | 514  (2%) | 1061  (5%) | 333  (2%) | 372  (2%) | 799  (4%) | 379  (2%) | 255  (1%) |

Table 6. The absolute number of readmissions stratified by the top ten diagnoses for readmissions between 2000 and 2013. The proportion of readmissions with each diagnosis are also presented in brackets. Fever was defined as ICD-10 code R50.9. GOR=gastro-oesophageal reflux (with ICD-10 codes K21.9 and K21.0). Cough, wheeze or shortness of breath defined as ICD-10 code R05X, R06.2, R06.6 or R06.8

|  | total of all readmission  (% with ten most common diagnoses) | Gastroenteritis | URTI/viral infection | Bronchiolitis | Cough, wheeze or shortness of breath | Asthma | Constipation | GOR | Febrile convulsion | Fever | Croup |
| --- | --- | --- | --- | --- | --- | --- | --- | --- | --- | --- | --- |
| 2000 | 1788  (34%) | 149  (8%) | 94  (5%) | 93  (5%) | 92  (5%) | 92  (5%) | 45  (3%) | 44  (2%) | 40  (2%) | 28  2%) | 18  (1%) |
| 2001 | 1951  (32%) | 149  (8%) | 88  (5%) | 114  (6%) | 112  (6%) | 80  (4%) | 42  (2%) | 35  (2%) | 54  (3%) | 48  (2%) | 25  (1%) |
| 2002 | 2143  (32%) | 188  (9%) | 98  (5%) | 172  (8%) | 115  (5%) | 82  (4%) | 37  (2%) | 34  (2%) | 63  (3%) | 41  (2%) | 30  (1%) |
| 2003 | 2695  (28%) | 156  (6%) | 138  (5%) | 156  (6%) | 130  (5%) | 64  (2%) | 71  (3%) | 28  (1%) | 63  (2%) | 53  (2%) | 50  (2%) |
| 2004 | 3140  (29%) | 189  (6%) | 133  (4%) | 184  (6%) | 200  (6%) | 127  (4%) | 80  (3%) | 33  (1%) | 57  (2%) | 50  (2%) | 30  (1%) |
| 2005 | 3416  (31%) | 251  (7%) | 134  (4%) | 165  (5%) | 239  (7%) | 135  (4%) | 70  (2%) | 38  (1%) | 70  (2%) | 56  (2%) | 63  (2%) |
| 2006 | 3665  (33%) | 270  (7%) | 158  (4%) | 246  (7%) | 269  (7%) | 167 (5%) | 68  (2%) | 57  (2%) | 83  (2%) | 85  (2%) | 39  (1%) |
| 2007 | 3724  (31%) | 255  (7%) | 161  (4%) | 297  (8%) | 246  (7%) | 125  (3%) | 61  (2%) | 60  (2%) | 73  (2%) | 89  (2%) | 90  (2%) |
| 2008 | 3380  (34%) | 239  (7%) | 190  (6%) | 336  (10%) | 292  (9%) | 126  (4%) | 43  (1%) | 67  (2%) | 65  (2%) | 79  (2%) | 43  (1%) |
| 2009 | 3379  (35%) | 267 (8%) | 149  (4%) | 245  (7%) | 313  (9%) | 116  (3%) | 59  (2%) | 52  (2%) | 75  (2%) | 98  (3%) | 65  (2%) |
| 2010 | 3224  (32%) | 256  (8%) | 169  (5%) | 281 (9%) | 239  (7%) | 103  (3%) | 59  (2%) | 69  (2%) | 51  (1%) | 71  (2%) | 30  (1%) |
| 2011 | 3566  (30%) | 247  (7%) | 156  (4%) | 341  (10%) | 209  (6%) | 123 (3%) | 56  (2%) | 80  (2%) | 43  (1%) | 75  (2%) | 72  (2%) |
| 2012 | 3653  (31%) | 288  (8%) | 215  (6%) | 395  (11%) | 180  (5%) | 162  (3%) | 55  (2%) | 73  (2%) | 45  (1%) | 52  (1%) | 49  (1%) |
| 2013 | 3833  (30%) | 209  (5%) | 223  (6%) | 350  (9%) | 152  (4%) | 134  (3%) | 89  (2%) | 95  (2%) | 54  (1%) | 64  (2%) | 143  (4%) |

Table 7. The mean and median duration of stay and proportion of admissions for zero, one, two, three and more than three days for each calendar year.

|  | Mean duration of stay (days) | Median duration of stay (days) | Proportion with admission for <1 day | Proportion with admission for 1 day | Proportion with admission for 2 days | Proportion with admission for 3 days | Proportion with admission for >3 days |
| --- | --- | --- | --- | --- | --- | --- | --- |
| 2000 | 1.70 | 1 | 25% | 37% | 16% | 8% | 13% |
| 2001 | 1.60 | 1 | 28% | 37% | 15% | 8% | 12% |
| 2002 | 1.47 | 1 | 34% | 34% | 14% | 7% | 11% |
| 2003 | 1.38 | 1 | 36% | 34% | 13% | 7% | 11% |
| 2004 | 1.30 | 1 | 39% | 32% | 12% | 6% | 10% |
| 2005 | 1.24 | 1 | 42% | 32% | 12% | 6% | 9% |
| 2006 | 1.19 | 1 | 43% | 31% | 1% | 6% | 9% |
| 2007 | 1.13 | 1 | 46% | 30% | 11% | 6% | 8% |
| 2008 | 1.11 | 1 | 47% | 29% | 11% | 6% | 8% |
| 2009 | 1.08 | 1 | 47% | 30% | 10% | 5% | 7% |
| 2010 | 1.09 | 1 | 47% | 30% | 10% | 6% | 8% |
| 2011 | 1.11 | 1 | 46% | 30% | 11% | 6% | 8% |
| 2012 | 1.08 | 1 | 47% | 30% | 10% | 5% | 7% |
| 2013 | 1.01 | 1 | 50% | 29% | 10% | 5% | 7% |

Table 8. The number of children with more than one admission in a calendar month with any diagnosis and with the same diagnosis.

|  | All admissions | Readmission with any diagnosis | Readmission with the same diagnosis | % with any readmission | % readmitted with same diagnosis | Scottish paediatric population | Readmission with any diagnosis/1000 | Readmission with the same diagnosis/1000 |
| --- | --- | --- | --- | --- | --- | --- | --- | --- |
| 2000 | 33305 | 1788 | 861 | 5.37 | 2.59 | 919439 | 1.94 | 0.94 |
| 2001 | 34097 | 1951 | 950 | 5.72 | 2.79 | 904997 | 2.16 | 1.05 |
| 2002 | 34308 | 2143 | 1118 | 6.25 | 3.26 | 890242 | 2.41 | 1.26 |
| 2003 | 35504 | 2695 | 1629 | 7.59 | 4.59 | 877685 | 3.07 | 1.86 |
| 2004 | 37467 | 3140 | 1885 | 8.38 | 5.03 | 871907 | 3.60 | 2.16 |
| 2005 | 40042 | 3416 | 1922 | 8.53 | 4.80 | 865091 | 3.95 | 2.22 |
| 2006 | 43927 | 3665 | 2083 | 8.34 | 4.74 | 856083 | 4.28 | 2.43 |
| 2007 | 44635 | 3724 | 1996 | 8.34 | 4.47 | 851334 | 4.37 | 2.34 |
| 2008 | 44744 | 3380 | 1701 | 7.55 | 3.80 | 850206 | 3.98 | 2.00 |
| 2009 | 44720 | 3379 | 1555 | 7.56 | 3.48 | 850477 | 3.97 | 1.83 |
| 2010 | 41325 | 3224 | 1533 | 7.80 | 3.71 | 851621 | 3.79 | 1.80 |
| 2011 | 43204 | 3566 | 1690 | 8.25 | 3.91 | 853891 | 4.18 | 1.98 |
| 2012 | 46139 | 3653 | 1817 | 7.92 | 3.94 | 853009 | 4.28 | 2.13 |
| 2013 | 46986 | 3833 | 1877 | 8.16 | 3.99 | 868921 | 4.41 | 2.16 |
